# Deception detection, transmission, and modality in age and sex

**DOI:** 10.3389/fpsyg.2014.00590

**Published:** 2014-06-13

**Authors:** Charlotte D. Sweeney, Stephen J. Ceci

**Affiliations:** ^1^Union Theological Seminary, New YorkNY, USA; ^2^Department of Human Development, Cornell University, IthacaNY, USA

**Keywords:** deception detection, pro-social lies, modality, older adults, college students

## Abstract

This study is the first to create and use spontaneous (i.e., unrehearsed) pro-social lies in an ecological setting. Creation of the stimuli involved 51 older adult and 44 college student “senders” who lied “authentically” in that their lies were spontaneous in the service of protecting a research assistant. In the main study, 77 older adult and 84 college raters attempted to detect lies in the older adult and college senders in three modalities: audio, visual, and audiovisual. Raters of both age groups were best at detecting lies in the audiovisual and worst in the visual modalities. Overall, college students were better detectors than older adults. There was an age-matching effect for college students but not for older adults. Older adult males were the hardest to detect. The older the adult was the worse the ability to detect deception.

## INTRODUCTION

Deception is defined as an individual’s deliberate attempt to convince another person to accept as true what the liar knows to be false, to gain some type of benefit or to avoid loss (Abe, 2011; Agosta et al., 2013). The ability to detect “micro expressions” (Ekman and Friesen, 1969), is a key skill for understanding a person’s true emotional state by detecting his or her quick, microsecond-long facial expression. However, these quick, facial expressions are often difficult to detect accurately and training results have been mixed (Hurley, 2012). Thus, lay people’s wishful thinking may lead them to believe that they can detect lies, despite ample research demonstrating that accuracy hovers around chance (Vrij, 2000; Edelstein et al., 2006; Bond and DePaulo, 2008).

The population aged 65 and older will more than double by 2050, rising from 39 million today to 89 million (United States Census Bureau, 2009). Thus, in sheer numbers alone, issues involving older adults will become increasingly relevant. Yet, very little is known about their willingness to engage in spontaneous lies to protect others or their skill in detecting lies of others.

In addition to the demographics of aging, it is important to study deception detection in older adults for four reasons. First, the need to detect deception in others is important in avoiding con artists for financial exploitation (Castle et al., 2012).

Second, it is important for older adults when serving as jurors to be able to detect deception in others. In Florida, from 2000 to 2006, adults aged 60 and over made up a significant proportion of jurors sitting on criminal juries (Ruva and Hudak, 2013).

Third, it is important to study deception detection in older adults because others may need to detect deception in them, especially in cases of elder abuse. Caregiver stress is the most often cited factor leading to elder mistreatment (McGreevey, 2005). Abused elders may lie about their abuse to avoid being placed in a nursing home, which is a legitimate concern (Lachs et al., 2002), or to avoid negative repercussions from an abusive caregiver (Davis and Medina-Ariza, 2001; Pillemer et al., 2011).

Finally, it is critical to study deception detection in older adults because they may also be party to criminal acts of deception, even though they are infrequent perpetrators. Crime in older adults does not significantly contribute to the nation’s crime rate, but as the older adult population has increased, so too have older adult crimes (Flynn, 2000). Some assume that older adults are less likely to offend because of the physical effects of aging (Flynn, 1996), but with the Internet, committing some forms of fraud and sex offenses are not as physically challenging. Although older generations may not use the Internet much at present, their Internet skills are expected to become more advanced.

In the deception detection literature very few studies have examined older adults’ abilities to both “send” and detect (“rate”) deception and, of those studies that have been conducted, the results are conflicting. For example, Bond et al. (2005) found that older adult females were more accurate at detecting deception than older males and younger adults of either sex, whereas Stanley and Blanchard-Fields (2008) found that older adults were worse at deception detection than younger adults and no gender difference was found. The different findings between these studies may result from differences in the methods and design, a key difference being that Bond et al. (2005) had both older adults and younger adults rate only younger adults – i.e., they had no conditions in which older adults rated other older adults and younger adults rating older adults. Stanley and Blanchard-Fields (2008) had older adults and college students only rate young males. Thus, it is important to have both age groups (older and college) rate their own age group as well as the other age group. Likewise, to determine whether there are age-matching effects and sex differences, it is necessary to have both males and females rate both their own sex and the other sex (necessitating a fully crossed design for both age groups and sexes).

Recently, Ruffman et al. (2012) examined younger and older participants’ judging the truthfulness of other younger and older speakers’ opinions. All participants found it easier to judge when an older adult was lying relative to a younger adult, and older adults were worse than young adults at detecting when speakers were telling the truth vs. lying. Neither younger nor older adults were advantaged when judging a speaker from the same age group. Overall, older adults were more transparent as liars and were worse at detecting rehearsed (i.e., not spontaneous or authentic) lies, with older adults’ worse emotion recognition mediating the relation between age group and lie detection failures.

Another area of unsettled science concerns the modality in which adults attempt to discriminate truthful from deceptive communications. Stanley and Blanchard-Fields (2008) showed younger and older adults interviews in one of three modalities: visual, audio, or audiovisual. They found that reduced emotion recognition in older adults was related to poor deception detection in the visual condition for the crime interviews only. Thus, it becomes of interest to use modality as a within-subjects variable whereby each participant receives all three modalities, not just one. Perhaps older adults are “captured” more by visual information than other age groups, something heretofore not examined.

Being able to match information from faces, voices, and bodies may be key to detecting deception. The inability to detect a mismatch between what a person says and his or her body language may be problematic in detecting deception, and here is where older adults, due to physical changes, may be most susceptible to deception (Castle et al., 2012).

When comparing younger and older adults’ ability to recognize and match body and facial expressions to vocal expressions, Ruffman et al. (2009) found that older adults were worse than younger adults in recognizing anger, sadness, fear, and happiness in body expressions as well as recognizing anger in vocal expressions.

Also, because younger adults tended to pay more attention to the eyes than older adults it has been suggested that this is why older adults are worse at recognizing sad, angry, and fearful expressions (Murphy and Isaacowitz, 2010). Older adults perform as well as younger adults on tasks where congruent auditory and visual emotional information are presented concurrently (Hunter et al., 2010). Again, this strongly suggests the value of adding modality as a within-subject variable, fully crossed with age as a between-subjects factor.

Finally, neural changes with aging have led some researchers to argue that multisensory information enhances older adult performance and may be a compensatory strategy for age-related reduction of brain activity in the sensory cortex (Cabeza et al., 2002; Peiffer et al., 2007; Hunter et al., 2010). Also, women tend to integrate multisensory emotional stimuli more efficiently than do men (Collingnon et al., 2010; Hunter et al., 2010; Ruffman et al., 2010). Thus age and sex differences in ability to detect deception should be examined.

In classic deception detection paradigms, participants are instructed (and rehearsed) to lie or pretend. This minimizes ecological validity because telling senders to lie may lessen their anxiety or guilt given that the lie is socially sanctioned. Thus, pretense or lying to conform to the experimenter’s instructions may lessen the emotional leakage cues of the sender, making it unnaturally harder for a rater to detect a lie in the sender. In some real-world instances of lying the sender has a great deal of anxiety, which is absent in contrived situations. The present study addresses this problem by motivating people to lie without ever asking them to do so. It accomplishes this, as will be seen, by creating pressures on the sender to lie to protect a novice research assistant from getting fired for making coding errors.

The present study examines deception detection within and between age groups in a fully crossed design. It explores within-subject age differences in deception detection based on modality (audio, visual, and audiovisual), and senders and raters of both sexes are enlisted to examine gender differences and gender × age × modality interactions. As noted, in this study senders were not asked to lie, but in some cases they spontaneously lied or told the truth on their own volition, adding a missing element in most lie detection research. This study tests participants (both senders and raters) in settings in which they are familiar. Older adults were tested in their own homes. Both the testing setting and the freedom to lie or tell the truth were assumed to add to the present study’s ecological validity. In addressing the four hypotheses of interest, visual acuity and verbal intelligence of both senders and raters were examined.

### HYPOTHESIS 1

Because older adults may not benefit from visual information to the same extent as younger adults (Stanley and Blanchard-Fields, 2008; Ruffman et al., 2009; Hunter et al., 2010), it is anticipated that their deception detection accuracy would be equivalent to college students only in the audio and the audiovisual conditions.

### HYPOTHESIS 2

Although one might expect peers to be more accurate in reading each other’s expressions because people have more experience with their own age group, based on previous research (Ruffman et al., 2012) no age-matching effect is expected in the current study.

### HYPOTHESIS 3

College students should be better at detecting deception than older adults (Stanley and Blanchard-Fields, 2008) since older adults have worse emotion recognition (Ruffman et al., 2012).

### HYPOTHESIS 4

Women should be better than men at detecting deception (Bond et al., 2005) and one reason might be their ability to integrate multisensory emotional stimuli more efficiently than men (Hunter et al., 2010).

Previous literature was extended by examining lies that were spontaneous (i.e., not prompted by the experimenter and unrehearsed) and of a pro-social nature. Such types of lies map more closely onto everyday life and since older adults were interviewed in their own homes this increased ecological validity.

## STIMULUS MATERIALS AND METHODS

### PARTICIPANT SENDERS

The goal of the pilot study was to develop a balanced stimulus set of participant senders for the main study of deception detection. Thus, results found from the pilot study should not be generalized beyond the pilot study.

Fifty-one older adults (31 females, 20 males) ranging in age from 65 to 86 years (*M* = 68.57, SD = 4.69) were from upstate New York and were recruited from a list of participants provided by the Cornell Institute for Translational Research on Aging (CITRA). Participants on this list were prescreened to exclude individuals with dementia and other memory and psychological health issues. Older adults received $20 for participating in the study.

Forty-four college students (25 females, 19 males) ranging in age from 18 to 23 years (*M* = 19.82, SD = 1.27) were recruited from two university campuses in upstate New York. All college students were recruited from majors outside of human development and psychology to minimize raters in the main study from recognizing participant senders in the video clips. College students received $10–20 for participating in the study. Participant accrual continued for both age groups until a balanced set of stimuli was obtained.

### PROCEDURE

To increase ecological validity, data were collected in an environment that participants would feel comfortable in and perceive as familiar. To this end, students were interviewed in a campus setting and older adults were given the option to be interviewed in their homes or on campus.

Upon arrival, participants were provided with a cover story indicating that a prospective research assistant was going to conduct a *practice* interview and would later be evaluated by a supervisor according to his or her accuracy and interpersonal skills. Participants gave informed consent to the specific activities involved, including consent to videotaping.

After video recording began and the research supervisor had left the room, the research assistant initiated a closely scripted conversation in which he or she disclosed a strong need to obtain the research position. Next, the assistant asked a series of demographic questions and recorded the participants’ answers. In repeating back participants’ answers, the assistant made a series of preordained mistakes.

Next, the research supervisor entered the room. She first asked the participant to describe the assistant’s interpersonal skills, nervousness, and ability to put the participant at ease. She then stated that her decision to hire the assistant would depend on the number of mistakes being two or fewer. She asked the participant whether the mistakes made by the assistant had been below that threshold.

After this key question had been answered, the research supervisor left the room and the research assistant fully debriefed the participant. Next, the research assistant completed a memory check by asking the participants to specify on which questions the research assistant had made mistakes. If the number of recalled mistakes differed from the number that the participant had admitted to the supervisor, the assistant also asked the participant: “For clarification purposes did you protect me by lying about the number of mistakes I made?” After this question was asked the research supervisor entered the room and asked the participant if he or she had any further questions that the research assistant may not have been able to answer during the debriefing.

## RESULTS

### DESCRIPTIVE ANALYSIS, STIMULUS SELECTION, AND CHARACTERISTICS

Since the research assistant made three planned mistakes, a participant was coded as having told the truth if she or he said the assistant made three mistakes. If a participant indicated that the assistant made two or fewer mistakes the participant was asked the clarifying question. If the participant said she or he lied to protect the assistant then that participant was coded as having made a pro-social lie. This is a conservative estimate given that some participants who lied probably endeavored to hide it by lying again.

To be categorized as having told the truth, the participant had to either state that three mistakes had been made or, if they failed to correctly remember the mistakes, state the number of mistakes that she or he remembered (as verified in the memory check and the discussion following the memory check). To be categorized as having lied a participant had to fit the following three criteria: (1) state that fewer than three mistakes had been made, (2) accurately remember that three mistakes had been made, and (3) state that she or he did protect the assistant by lying about the number of mistakes. When categorizing lies vs. truths, a participant could be classified in one of three categories: lied, told the truth, or was not classifiable due to either memory problems, contradictory statements made by the participant, or experimenter error. Clips that were not classifiable were not included in the 24 clips to be viewed by raters in the main study.

Twenty-four clips were selected, with three clips in each age (older vs. college student), gender (male vs. female), and veracity category (truth vs. lie). The average length of the clips was in minutes:seconds.milliseconds: *M* = 1:24.37, with a range from 0:51.23 to 2:17.98, SD = 0:23.00 s.

The clips showed the participant sender from the head to the knees. The research assistant and the research supervisor could be heard but not seen.

## MATERIALS AND METHODS OF MAIN STUDY

### PARTICIPANTS

Seventy-seven older adults (38 females, 39 males), age range = 60 to 93, *M* = 73.77, SD = 7.79 were recruited from upstate New York, as described in the stimulus design of the Pilot Study. Older adult raters were paid $20 for participating in the study.

Eighty-four college students (41 females, 43 males), age range = 18 to 23, *M* = 19.58, SD = 1.25 were recruited from two university campuses in upstate New York and they received 1–2 course credits that were applied to their psychology course work. All raters (both older and college) were Caucasian so as not to introduce race as one of the variables at this time.

### DESIGN

The study design was a 2(rater age: older adult, college student) × 2(sender age: older adult, college student) × 2(sex of sender) × 2(sex of rater) × 3(modality: audio, visual, audiovisual) × 2(condition: truth vs. lie) mixed model design. Rater age was a between-subject variable, the age and sex of the sender in the video clip were within-subject variables. Modality and truth–lie condition were within-subject variables. Subject was a random factor. Each rater rated all 24 clips, which were presented randomly regarding modality, sex, age, and truth value. Half the senders in the clips were lying, but raters were not told this, since this information could lead to the use of inferential strategies instead of lie detection skills (Leach et al., 2004). For each clip raters were asked for a binary (truth/lie) response as well as a 5-point Likert scale for confidence.

### STIMULI

Twenty-four audiovisual clips were created to show realistic instances of lying or truth-telling split by age group (college aged vs. older adult) and gender (see Pilot Study).

### PROCEDURE

Older adult and college student raters were asked to determine whether the person in each video clip was lying or telling the truth. Showing all 24 clips took 30 min for college raters and 45 min for older adult raters. In addition, all raters were asked demographic questions (age, race, country of origin, years of education or highest degree attained, occupation prior to retirement, if retired). Modality of clip was presented randomly. In total, a third of all clips were audio, another third were visual, and another third were audiovisual.

Raters were shown two sample test clips. The first clip was used to test for vision and hearing. No raters complained about having trouble seeing, since they were invited to move the laptop wherever they could best see. Raters who were hard of hearing wore a headset. The second clip was an example of a typical clip, so raters would know what to expect and could ask any questions before viewing the actual clips. Raters were shown the clips, while an assistant recorded the raters’ responses and the length of time it took for the rater to make a response. Then raters were asked how confident they were of their truth–lie response on a five-point Likert scale with 1 = very unsure, to 5 = very sure.

### MEASURES

Due to time constraints on the young adults, it was not possible to include all of the measures that were employed with older adults, such as the *Mini-Mental State Exam* and the *WAIS-R* vocabulary. Years of education are as follows: college (*N* = 83, *M* = 13.31, SD = 1.09) and older adults (*N* = 77, *M* = 17.19, SD = 3.68).

#### Modified Mini-Mental State Exam

All older adults took a modified six question version of the *Modified Mini-Mental State Exam* to screen for dementia and memory impairment (Folstein et al., 1975; Mueller-Johnson and Ceci, 2004). Some of the college students were given the *Modified Mini-Mental State Exam* to see how they compared with older adults, but it can be safely assumed that to perform at a competitive, select college these students did not have dementia or memory impairment. The cut-off for the *Modified Mini-Mental State Exam* inclusion for being in the study was scoring at least 3 out of 6. No one received less than a 3 out of 6 (older adults: *N* = 77, *M* = 5.75, SD = 0.52; college: *N* = 36, *M* = 5.86, SD = 0.42).

#### WAIS-R vocabulary

The highest a participant could score on the WAIS (Wechsler, 1981) was 70 (older: *N* = 77, *M* = 58.34, SD = 6.02; college: *N* = 11, *M* = 53.64, SD = 4.95).

#### Snellen test for visual acuity

All older raters and some college raters were given the *Snellen Visual Acuity test*. For older adult raters the mean corrected vision score was 20/30 and for college raters the mean corrected vision score was 20/20. Raters were asked about the amount of time they spent with their own age group and the other age group.

#### Generalized estimating equations (GEE)

In addition to binary logistic regression, generalized estimating equations (GEE) were used to model accuracy in detecting both truths and lies. GEE provides percentages for accuracy, where chance is 50%. Since each rater saw all 24 clips, the data were clustered such that each rater was his or her own cluster. GEE uses a simple correction of the estimated SEs to account for the within-cluster correlation (Norton et al., 1996).

#### Signal detection analysis

Signal detection has an advantage over analyses based on mean accuracy alone because it allows one to break down raters’ decisions into two parts: (1) the rater’s ability to discriminate between truth- and lie-tellers (*d*′), and (2) a measure of rater’s response bias *C* (Leach et al., 2004).

In this study, a *hit* was a correct detection of a lie. A *false alarm* was when a rater thought a sender was lying, when the sender was really telling the truth. In signal detection there are two probabilities, the hit rate, which is the number of hits divided by the number of signal (lie) trials, and the false alarm rate, which is the number of false alarms divided by the number of noise (truth) trials (Wickens, 2002). Neither the hit rate nor the false alarm rate is sufficient on its own. A single number that represents the rater’s sensitivity to the signal is best. This number is represented by discriminability (*d*′).

Discriminability (*d*′) is estimated: *Z*_hits_ - *Z*_false alarms_, which is a measure that corrects for response bias and for guessing (Bond et al., 2005). *Z*_hits_ represent the z score of the number of lie decisions that were made when lies were present in the clips. *Z*_false alarms_, represents the number of lie decisions made when truthful statements were present in the clips.

The false alarm rate and the noise distribution are used to estimate the criterion *C*, or participant bias with *C* defined as the distance of the criterion from the intersection of the two underlying distributions (Snodgrass and Corwin, 1988).

(1) $$C=Z_{false\,\;alarms}-d\prime /2$$

(The larger the *d*′, the better the discriminability. When the *C*-value is 0, this indicates no bias. When assessing lie as the signal, a negative *C*-value indicates a truth-bias and a positive *C*-value indicates a lie-bias.)

## RESULTS

Binary logistic regression and GEE were used to calculate the estimated marginal means (EMMeans) to demonstrate pairwise comparisons. **Table 1** shows the full model and **Table 2** shows the final model for those variables and interactions that were significant or meaningful. For all pairwise comparisons Bonferroni corrections were made.

**Table 1:** Full generalized estimating equations model.

Variable	Generalized score chi-square	df	*p*
Sender age	11.641	1	0.001
Sender sex	8.527	1	0.003
Rater age	19.319	1	<0.001
Modality	61.914	2	<0.001
Sender age × sender sex	43.344	1	<0.001
Sender age × rater age	2.973	1	0.085
Sender age × modality	10.090	2	0.006
Sender sex × rater age	0.029	1	0.865
Sender sex × modality	1.249	2	0.536
Rater age × modality	12.838	2	0.002
Sender age × sender sex × rater age	0.268	1	0.605
Sender age × sender sex × modality	1.871	2	0.392
Sender age × rater age × modality	0.996	2	0.608
Sender sex × rater age × modality	0.143	2	0.931
Sender age × sender sex × rater age × modality	3.492	2	0.174

**Table 2:** Test of model effects (generalized estimating equations).

Variable	Generalized score chi-square	df	*p*
Sender age	11.256	1	0.001
Sender sex	8.200	1	0.004
Rater age	17.945	1	<0.00
Modality	45.185	2	<0.00
Sender age × sender sex	34.595	1	<0.001
Sender age × modality	9.679	2	0.008
Rater age × modality	12.321	2	0.002
Sender age × rater age	3.230	1	0.072

As discussed in the measures section GEE provides percentages for accuracy (the outcome variable) where chance is 50%. Our predictor variables were: rater age, rater sex, sender age, sender sex, and modality.

### HYPOTHESIS 1: OLDER ADULTS RATING EITHER AGE GROUP WILL DO EQUALLY WELL IN THE AUDIO AND AUDIOVISUAL CONDITIONS

#### Modality

**FIGURE 1:**
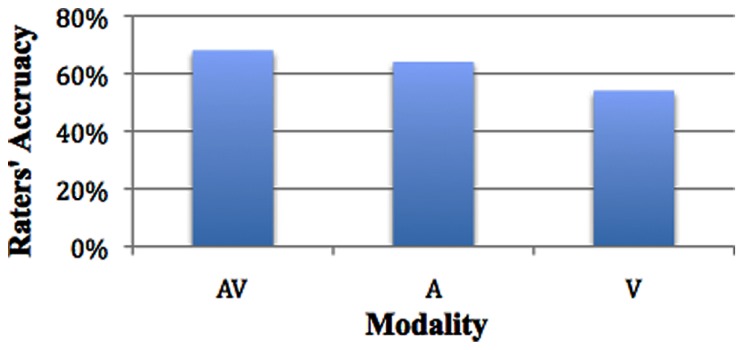
**Accuracy across modality**.

Estimated marginal means for modality show mean accuracy for audiovisual to be 68% (SE = 1.4%); mean accuracy for audio to be 64% (SE = 1.4%); and mean accuracy for visual to be 54% (SE = 1.3%), χ^2^_(2)_ = 63.029, *p* < 0.001. There was no significant difference between audiovisual (68%) audio (65%), SE = 1.8%, however, audio (65%) was significantly more likely to lead to greater accuracy than visual (54%), SE = 1.8%, *p* = 0.000, and audiovisual (68%) was significantly more likely to increase accuracy than visual (54%), SE = 1.9%, *p* < 0.001 (see **Figure 1**).

#### Raters’ age and modality

**FIGURE 2:**
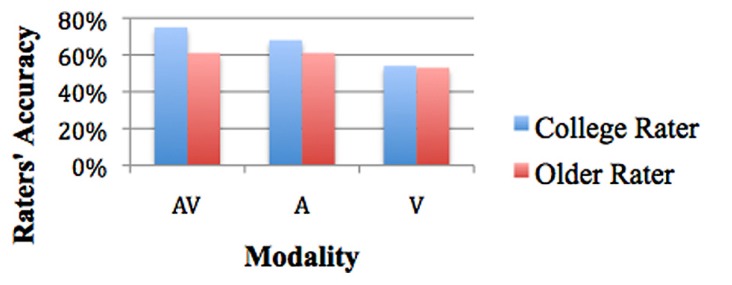
**Rater’s age and accuracy by modality**.

Estimated marginal means show that in the audiovisual modality college rater accuracy was 75% (SE = 1.8%) and older rater accuracy was 61% (SE = 2.2%). In the audio modality college rater accuracy was 68% (SE = 1.8%) and older rater accuracy was 61% (SE = 2.1%). In the visual modality college rater accuracy was 54% (SE = 1.8%) and older adult accuracy was 53% (SE = 1.8%; see **Figure 2**).

The overall chi-square of these pairwise comparisons revealed significant differences for the six conditions, χ^2^_(5)_ = 113.226, *p* < 0.001. College raters were significantly better at accuracy in the audiovisual modality (75%) than in the visual modality (54%), SE = 2.4%, *p* < 0.001. College raters were significantly more accurate in the audio modality (68%) than in the visual modality (54%), SE = 2.2%, *p* < 0.001. College raters were only marginally more accurate in the audiovisual modality (75%) than in the audio modality (68%), SE = 2.4%, *p* = 0.063. Thus, college raters’ accuracy declined when audio was removed (see **Figure 2**).

Older raters were not significantly different in either the audio modality (61%) or in the audiovisual modality (61%), SE = 2.5%, *p* = NS. Older raters were marginally better in the audiovisual modality (61%) than in the visual modality (53%), SE = 2.8%, *p* = 0.072. Older raters were marginally better at accuracy in the audio modality (61%) than in the visual modality (53%), SE = 2.7%, *p* = 0.063. Thus, older raters did equally well in the audiovisual and audio modalities and showed a marginal decline when audio was removed (see **Figure 2**).

#### Comparing modality between age groups

In the audiovisual modality, college raters were more accurate (75%) than older raters (61%), SE = 2.8%, *p* < 0.001. In the audio modality, college raters were more accurate (68%) than older raters (61%), SE = 2.7%, *p* = 0.045, whereas in the visual modality, there was no difference between college rater (54%) and older rater (53%) accuracy, SE = 2.5%, *p* = NS. Thus, college raters gained more from combining the audiovisual modality than older raters (see **Figure 2**).

### HYPOTHESIS 2: AGE-MATCHING EFFECTS

**FIGURE 3:**
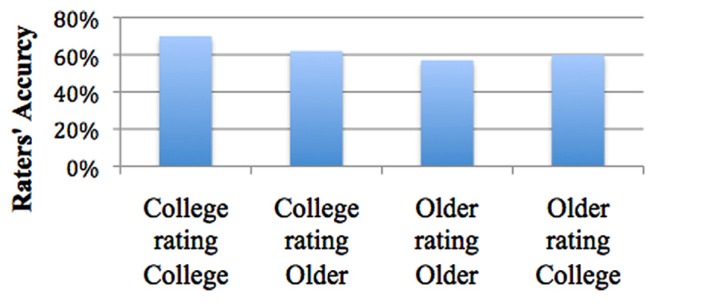
**Age-matching effects**.

Mean accuracy rate for college rating other college students was 70% (SE = 1.6%), while mean accuracy rate for older rating older was 57% (SE = 1.6%). Mean accuracy for college rating older were 62% (SE = 1.7%), while mean accuracy rates for older rating college students were 60% (SE = 1.6%; see **Figure 3**).

There was an age-matching effect for college students but not for older adults. The difference in mean accuracy between college students rating college senders (70%) and older participants rating older senders (57%) was significant, 0.13, SE* = *2.3%, *p* < 0.001. Thus, college raters were significantly better at rating their peers than older raters were at rating their own peers. The difference in accuracy between college students rating other college students (70%) and older raters rating college students (60%) was also significant, SE* = *2.3%, *p* < 0.001. And finally, the difference in mean accuracy between college students rating college students (70%) and college students rating older adults (62%) was significant, SE = 2.3%, *p* = 0.004. College raters were better at rating their peers than they were at rating older adults. However, the difference in accuracy between college rating older (62%) and older rating older (57%) was not significant. In sum, it was challenging for both rater age groups to accurately detect older adult senders (see **Figure 3**).

### SIGNAL DETECTION: MODALITY

Linear mixed models were used to control for repeated measures, since there were three different modalities (audio, visual, and audiovisual) for each rater. EMMeans were used to compute pairwise comparisons. For each test, the dependent measures were individual *d*′ and *C* parameters. **Table 3** shows the final model for *d*′ when lie was the signal.

**Table 3:** Signal detection analysis final model of *d*′ (discriminability) when lie was the signal.

Source	Numerator df	Denominator df	*F*	*P*
Modality	2	316	28.516	<0.001
Raters sex	1	157	0.459	0.499
Rater age	1	157	9.036	0.003
Modality × rater age	2	316	3.723	0.025

Pairwise comparisons for modality within each age group for mean *d*′ scores were conducted. College raters were significantly better at discrimination in the audio modality (*M* = 2.803) than in the visual modality (*M* = 0.542), SE = 0.457, *p* < 0.001; they were also significantly better at discrimination in the audiovisual modality (*M* = 3.757) than in the visual modality (*M* = 0.542), SE = 0.457, *p* < 0.001. Older raters were also significantly better at discrimination in the audio modality (*M* = 2.065) than the visual modality (*M* = 0.459), SE = 0.457, *p* = 0.005 and were also better in the audiovisual modality (*M* = 1.895) than the visual modality (*M* = 0.459), SE = 0.457, *p* = 0.015. These results reveal that as auditory information was removed, both college and older raters became less accurate.

Next, mean differences in *d*′ scores were examined within each modality between the two age groups. In the audiovisual modality, college raters were significantly better at discrimination (*M* = 3.757, SE = 0.335) than older adults (*M* = 1.895, SE**= 0.348), *p* < 0.001. In contrast, in the audio modality, college raters were not significantly better (*M* = 2.803, SE = 0.335) than older adults (*M* = 2.065, SE = 0.348), *p* = NS. In the visual modality, college raters were not significantly better at discrimination (*M* = 0.542, SE = 0.335) than older adults (*M* = 0.459, SE = 0.348), *p* = NS. Thus, college raters were helped more than older raters in the audiovisual modality, but not more than older raters in either the audio or visual modalities alone. **Table 4** shows the final model for *C* bias when lie was the signal.

**Table 4:** Signal detection analysis final model of *C* bias for lie as signal.

Source	Numerator df	Denominator df	*F*	*P*
Modality	2	318	45.717	<0.001
Raters sex	1	157	0.102	0.750
Rater age	1	157	0.068	0.795

Raters showed a truth-bias for all three modalities: audiovisual *C* bias = -4.002, SE = 0.276, audio *C* bias = -3.925, SE = 0.276, and for visual *C* bias = -0.853, SE = 0.276. The only significant differences were: raters in the audiovisual modality showed a greater truth-bias than raters in the visual modality, SE = 0.376, *p* < 0.001; and raters in the audio modality showed a greater truth-bias than raters in the visual modality, SE**= 0.376, *p* < 0.001. The three-way interaction for *C* bias was not significant. There was no significant truth bias between audiovisual and audio, SE = 0.376, *p* = NS. Thus, any modality with an audio component led to a greater truth-bias than visual.

There was no significant difference in *C* bias for either rater sex (female raters’ *C* bias was -2.981, SE = 0.244; male raters’ *C* bias was -2.872, SE = 0.238) or rater age (college raters’ *C* bias was -2.971, SE = 0.237; older raters’ *C* bias was –2.883, SE = 0.246).

### HYPOTHESIS 3: COLLEGE STUDENTS ARE BETTER DETECTORS THAN OLDER ADULTS

**FIGURE 4:**
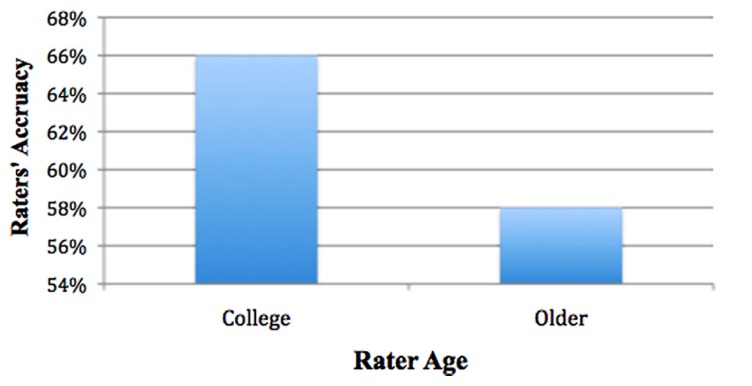
**College more accurate than older**.

Pairwise comparisons showed that indeed, college raters were overall significantly more accurate than older adult raters (66% for college, SE = 1.2%; 58% for older adult, SE = 1.3%), χ^2^_(1)_ = 20.123, *p* < 0.001). Signal detection analyses were conducted to examine age differences, collapsing across modality (see **Figure 4**).

#### Signal detection: age differences (not taking into account modality)

**Table 5** shows means and SDs for *d*′ and *C* bias when lie was the signal. For *d*′ overall college students were significantly better at detecting lies (college students had a significantly higher *d*′ discrimination value for lies: *M* = 1.05, SD = 1.10 vs. older adults: *M* = 0.56, SD = 1.08, *t*(158) = 2.82, *p* = 0.005).

**Table 5:** Signal detection analysis of means and SD for *d*′ and *C* for lie as signal.

	*d*′ (lie detection ability)		Bias *C* criterion	
Age group rater	*M*	SD	*M*	SD
College	1.05	1.10	-1.36	1.54
Older adult	0.56	1.08	-1.26	1.49

For *C* bias there were no significant differences between college (*M* = -1.36, SD = 1.54) and older adults (*M* = –1.26, SD = 1.49), *t*(158) = -0.41, *p* = 0.681, both of whom exhibited a truth bias.

#### Confidence and latency

GEE were employed to assess confidence and latency. (Signal detection could not be used because it would involve grouping the data by collapsing it into categories thus obscuring confidence rating and latency measures.) Both confidence and latency were categorized as continuous variables. The test of model effects for confidence and latency are shown in **Table 6**.

**Table 6:** Test of model effects: confidence and latency.

Variable	Generalized score chi-square	df	*p*
Confidence	0.015	1	0.901
Latency	0.045	1	0.832
Confidence × latency	5.291	1	0.021

The mean for confidence was 3.51, where 1 = very unsure and 5 = very sure. The mean for latency was 01:21.70, in minutes, seconds, and milliseconds, and this was the amount of time it took raters to make a truth / lie decision for each sender clip. The parameter estimates for confidence centered were *B* = 0.286, SE = 0.0355, Wald χ^2^ = 64.876, df = 1, *p* < 0.001. For latency centered, the parameter estimates were *B* = 0.011, SE = 0.0016, Wald χ^2^ = 45.053, df = 1, *p* < 0.001. The interaction between confidence centered and latency centered was *B* = 0.003, SE = 0.0013, Wald χ^2^ = 5.466, df = 1, *p* = 0.019. This means that raters who were more confident were more accurate and raters who took longer to make a decision were also more accurate.

#### Differences in latency between college student and older adult raters

College student raters had significantly shorter latencies (faster reaction times) than older adult raters, *F* = 12.217, *t*(3822) = -8.327, *p* < 0.001. Average latency for college student raters was *M* = 0.01:18, *N* = 1985, SD = 0.00:24, while average latency for older adult raters was *M* = 0.01:25, *N* = 1839, SD = 0.00:27, where latency was in minutes, seconds, and milliseconds.

#### Post hoc analyses

**FIGURE 5:**
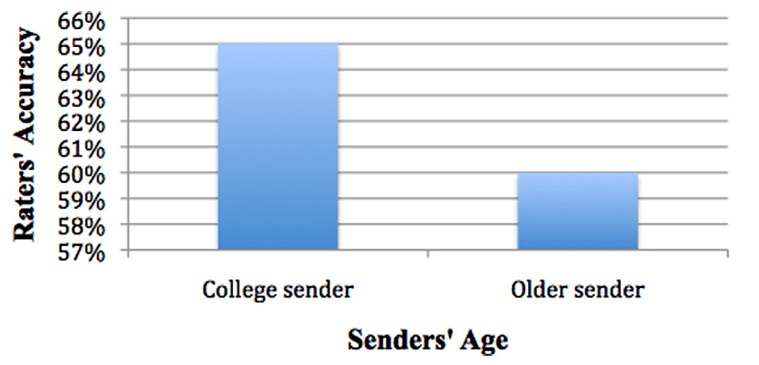
**College senders easier to detect than older**.

***Is one age group easier to detect than another?*** Pairwise comparisons show sender age was associated with significant differences in accuracy, with raters’ accuracy being higher when viewing college senders (*M* = 65%, SE = 1.2%) than when viewing older adults (*M* = 60%, SE = 1.2%), χ^2^_(1)_ = 12.1222, *p* < 0.001 (see **Figure 5**).

**FIGURE 6:**
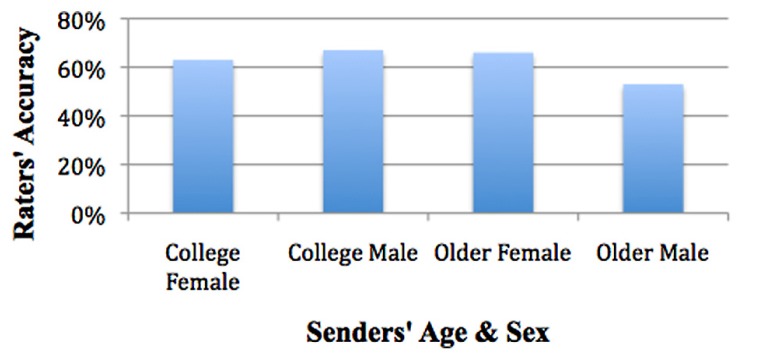
**Older males most difficult to detect**.

Estimated marginal means for senders’ age and senders’ sex showed college females’ accuracy was 63%, SE = 1.4%, college males was 67%, SE = 1.5%, older females was 66%, SE = 1.5%, and older males was 53%, SE = 1.4% (see **Figure 6**).

Mean accuracy for participants rating an older female (*M* = 66%) were significantly greater than for rating of an older male (*M* = 53%), SE = 1.8%, *p* = 0.000. There were no significant differences for participants rating a college male (*M* = 67%) vs. a college female (*M* = 63%), SE = 1.8%, *p* = NS. Mean accuracy for participants rating a college male (67%) were significantly greater than rating an older male (*M* = 53%), SE = 1.8%, *p* < 0.001. There were no significant differences for participants rating an older female (66%) compared to a college female (*M* = 63%), SE = 1.9%, *p* = NS; χ^2^_(3)_ = 71.491, *p* < 0.001. Older males were the most difficult for raters to detect (see **Figure 6**).

**FIGURE 7:**
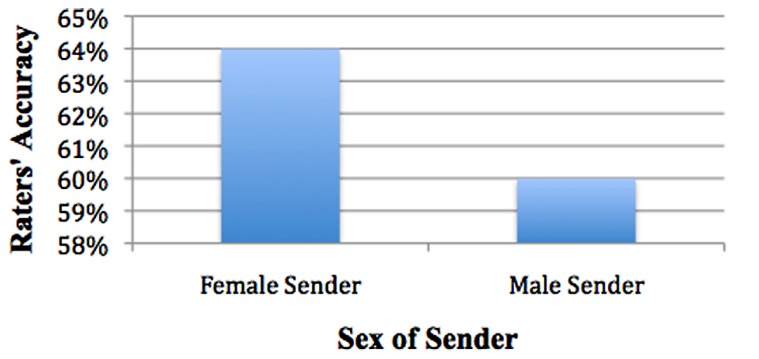
**Females easier to detect than males**.

***Is one sex easier to detect than the other?*** Using EMMeans and pairwise comparisons, sender’s sex led to significant differences in accuracy with rater’s accuracy being higher when viewing a female sender (64% accuracy rate, SE = 1.1%) than when viewing a male sender (60% accuracy rate, SE = 1.2%), χ^2^_(1)_ = 8.624, *p* = 0.003. Thus female senders were significantly easier to detect than male senders (see **Figure 7**).

***Are younger-older adults more accurate than older-older adults?*** A regression analysis with lie as the signal (*d*′) as the dependent variable and the numerical value of older adult raters’ age as the independent variable was conducted. The younger the older adult was the more accurate, or alternatively, the older the older adult was, the less accurate, *B* = -0.048, SE = -0.015; *t* = -3.200; *p *= 0.002.

***Time spent with own and other age group.*** Both older and college raters were asked how much time they spent interacting with college students and with older adults per year. Time spent with either college students or older adults did not predict discrimination ability.

## DISCUSSION

The goal of the present research was to provide a finer-grained examination of both the ability to detect lies and the willingness and skill in transmitting (sending) them. Toward this end, the interactive effects of age, gender and modality were assessed for a variety of dependent variables. It was hypothesized (***Hypothesis 1***) that older adults rating college students and other older adults would do equally well in deception detection in the audio and the audiovisual conditions because they may not benefit from visual information (or be able to integrate it) to the same extent as young adults (Stanley and Blanchard-Fields, 2008). Regardless of age, audio and audiovisual modalities led to greater accuracy than visual.

Older raters did equally well in the audio and audiovisual modalities. College raters were better at detecting lies both in the audiovisual and audio modalities than in the visual modality and they were helped to a greater extent in the audiovisual modality than were older raters.

In general, college students were more accurate than older adults and neither age group benefited from the visual modality. This supports Stanley and Blanchard-Fields’s (2008) finding that older adults were less accurate in the visual condition, and for both ages removing audio input reduced deception detection accuracy.

### HYPOTHESIS 2

There was an age-matching effect for college students but not for older adults. College students were significantly more accurate at detecting deception in their peers, while older adults were not. This goes against Ruffman et al.’s (2012) finding of no age-matching effect. One possible explanation for this difference was that older adults in Ruffman et al.’s (2012) study were asked to lie about their opinions. However, the present study senders were induced to lie spontaneously, without the encouragement of experimenters. As noted at the outset, data on deception detection is limited to the extent that it is based on sanctioned artifice rather than real-world spontaneous lies, which may entail far more emotional leakage, at least for some groups.

### HYPOTHESIS 3

College students were hypothesized to be better at detecting deception than older adults (Stanley and Blanchard-Fields, 2008), and this was supported, in the present findings. Although the study was not designed to explore the basis of their superiority, pervious research argued that older adults have worse emotion recognition (Ruffman et al., 2012), which may underpin their lower accuracy. In addition, college raters may have been better at deception detection than older raters because older adults may be experiencing neurological changes that make them more trusting of others than is warranted by circumstances (Castle et al., 2012). In addition, since audiovisual is so fundamental to accurate deception detection, it may be older adults’ weaker hearing and vision, even after correction, that may be contributing to this age difference in accuracy. In addition, a *post hoc* analysis showed that the older the raters were, the worse they were at detecting deception. This is consistent with physiological decrements in perception (given that there were no cognitive differences on the Modified Mini Mental State Exam).

No sex differences were found in ability to detect deception. Contrary to Bond et al. (2005), women were no better than men at deception detection (***Hypothesis 4***). College students’ lies were easier to detect than older adults’ and older adult males were the most difficult to detect (for details see *Post hoc* Analyses). Perhaps older males may have displayed less affect and may have given less verbal information than the other three groups (college females, college males, and older females), but this finding would require further testing. Females were easier to detect than males (see *Post hoc* Analyses). One possibility for this finding is that females may be more emotionally expressive in attempting to connect with the interviewers as some research suggests. Examining gender differences in emotional expression and communication styles might aid in the explanation of why older adult males were the hardest to detect and women the easiest to detect.

While the present study found older adult males the most difficult to detect, Ruffman et al. (2012) discovered just the opposite. They found older adults to be more transparent in their lying. Again, this difference in findings may have more to do with study design, the present study eliciting spontaneous lies and Ruffman et al.’s (2012) eliciting lies about opinions.

### TRUTH-BIAS AND AGE

Both college and older raters showed a comparable truth-bias. While raters showed a truth-bias for all three modalities, those in the audiovisual and audio modalities showed a greater truth-bias than raters in the visual modality. Raters of both ages and both sexes had the same level of truth bias when trying to detect lies, possibly, because most psychologically healthy people are trusting (American Psychiatric Association, 1994; Roth et al., 1997; Fenster and Fenster, 1998).

### CONFIDENCE

Previous studies have found that raters’ confidence in their truth / lie decisions are not a good indicator of accuracy (DePaulo and Pfeifer, 1986; Ekman and O’Sullivan, 1991; Vrij, 2000; Garrido et al., 2004). The current study, however, found that as confidence increased, so did accuracy. This may be due to the ecological validity of the senders’ lies, as they were not given permission to lie and presumably as a consequence when they did lie it was accompanied by greater emotional leakage. Telling senders to lie may lessen their anxiety or guilt about lying, because the culpability of the lie rests on the researcher, and thus the sender is less apt to “leak” emotional cues. Thus raters may have felt more certain in their truth / lie decisions, which increased their confidence.

One limitation of this study may be the difference in testing context for college students vs. older adults. While testing college students on campus and older adults in their homes was meant to increase ecological validity, these two testing contexts may have primed these two groups differently. The home may feel like a trusting environment, while the campus environment may prime critical thinking and competition. Testing older and younger adults in the same environment might work to equalize environmental priming. Another limitation of this study is that administering the Implicit-association-test (IAT) would have allowed us to determine whether there was a correlation between raters’ accuracy in detecting deception and raters’ attitudes regarding age and sex of senders.

In conclusion, since the population of adults aged 65 and older will more than double by 2050 in the United States (United States Census Bureau, 2009), it is important that psychological researchers understand their cognitive and sensory strengths and weaknesses. The present study was an attempt to provide data on a specific set of variables and it revealed a number of new findings.

## Conflict of Interest Statement

The authors declare that the research was conducted in the absence of any commercial or financial relationships that could be construed as a potential conflict of interest.
